# Key risk factors and adverse outcomes in metachronous vertebral osteomyelitis following periprosthetic joint infection: A 5‐year retrospective study

**DOI:** 10.1002/jeo2.12083

**Published:** 2024-07-05

**Authors:** Yu‐Chih Lin, An‐Jhih Luo, Fu‐Cheng Kao, An‐Shun Tai, Yuhan Chang, Pang‐Hsin Hsieh, Sheng‐Hsun Lee, Sheng‐Hsuan Lin

**Affiliations:** ^1^ Department of Orthopedic Surgery Chang Gung Memorial Hospital (CGMH) Kweishan Taoyuan Taiwan; ^2^ Bone and Joint Research Center, Chang Gung Memorial Hospital (CGMH) Kweishan Taoyuan Taiwan; ^3^ College of Medicine Chang Gung University (CGU) Taoyuan Taiwan; ^4^ Institute of Statistics National Chiao Tung University Hsinchu Taiwan

**Keywords:** clinical outcomes, metachronous vertebral osteomyelitis, periprosthetic joint infection

## Abstract

**Purpose:**

Periprosthetic joint infection (PJI) is a leading cause of joint arthroplasty failure, potentially leading to critical complications like vertebral osteomyelitis (VO). The factors contributing to VO after PJI and the outcomes for these patients are not well understood. Our study aims to (1) identify risk factors for VO following PJI and (2) assess the clinical outcomes in these cases.

**Methods:**

We included PJI patients treated surgically at our centre from January 2006 to December 2020, excluding those with simultaneous VO post‐PJI. Our focus was on patients with VO occurring after PJI, monitored for at least 5 years. Analysis included patient comorbidities, PJI treatment approaches, pathogen identification and clinical outcomes.

**Results:**

Of 1701 PJI cases, 21 (1.23%) developed VO. Key risk factors for VO post‐PJI were identified: systemic inflammatory response syndrome, substance misuse, polymicrobial infection and undergoing at least three stages of resection arthroplasty (odds ratios: 1.86, 54.28, 52.33 and 31.88, respectively). Adverse outcomes were noted in VO patients, with recurrent VO in 6/21 and repeated PJIs in 18/21 cases.

**Conclusions:**

Patients with PJI, especially those with certain risk factors, have an increased likelihood of developing VO and encountering negative outcomes. The potential role of bacteremia in the development of VO after PJI needs further exploration.

**Level of Evidence:**

Level III.

## Graphical abstract

Visual summary of key findings. This figure illustrates the major outcomes of our study, highlighting the incidence rate of metachronous vertebral osteomyelitis (VO) postperiprosthetic joint infection and the identified key risk factors contributing to VO development. The graph showcases the odds ratios for systemic inflammatory response syndrome, substance misuse, polymicrobial infection and multiple stages of resection arthroplasty, underlining their significant roles in VO occurrence. This summary aids in quickly understanding the study's impact on clinical practices.

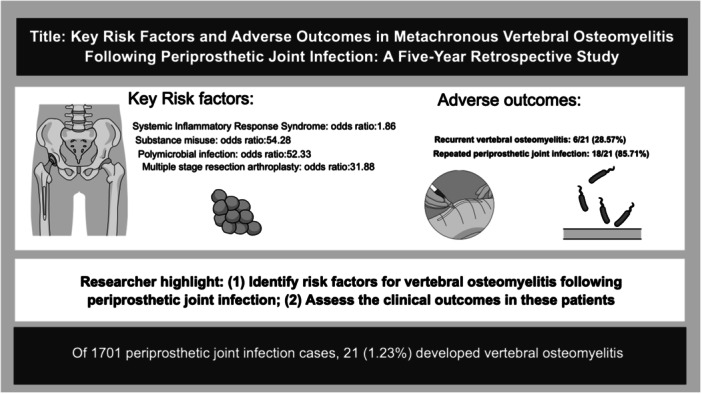

**Abbreviations**
ALBC: antibiotic‐loaded bone cement
CRP: C‐reactive protein
ESR: serum erythrocyte sedimentation rate
ICD‐9: International Classification of Diseases, Ninth Revision, Clinical Modification
IV: intravenous
OR: odds ratio
PJI: periprosthetic joint infection
SIRS: systemic inflammatory response syndrome
THA: total hip arthroplasty
TKA: total knee arthroplasty
VO: vertebral osteomyelitis

## INTRODUCTION

Recent advancements in orthopaedic implants have significantly enhanced surgical options and patient outcomes. There's a growing trend in joint replacement surgeries, especially among older adults, with the average age of these patients notably declining [4]. Additionally, there's an increase in individuals undergoing multiple joint replacement operations [18]. As these surgeries become more frequent, attention has shifted towards complications postarthroplasty, with periprosthetic joint infection (PJI) being the most critical, straining healthcare resources, incurring high costs and often leading to poor patient outcomes [9]. Treatment‐related complications in PJI cases are common [7].

Studies show that about 20% of PJIs are either metachronous or synchronous [8, 11, 13, 14, 23]. ‘Metachronous’ refers to infections occurring at different times in separate joints, while ‘synchronous’ refers to infections occurring simultaneously in different joints. There's also growing concern about metachronous vertebral osteomyelitis (VO) post‐PJI. VO accounts for 2%–7% of all musculoskeletal infections, with an increase in the last few decades [19], often necessitating spinal surgery when antibiotics fail.

The incidence and risk factors for metachronous VO after PJI are not fully explored in existing literature. The role of hematogenous spread in its pathogenesis is speculated but not confirmed. This study aims to identify potential risk factors, including patient comorbidities, PJI management techniques and causative pathogens and assess the clinical outcomes for patients developing VO following PJI.

## METHODS

### Data source

This research conducted a retrospective analysis on a group of patients diagnosed with PJI of the hip or knee, who received treatment at our specialised referral medical centre between January 2006 and December 2020. All methods adhered to relevant guidelines and regulations. The Institutional Review Board (IRB) waived the need for informed consent and provided authorisation for this study (IRB: 201601034B0).

#### Codes of interest

This study involved identifying patients who had undergone total hip arthroplasty (THA) or total knee arthroplasty (TKA) using International Classification of Diseases, Ninth Revision, Clinical Modification (ICD‐9‐CM) procedure codes (81.51, 81.53, 81.54, 81.55) and ICD‐10 procedure codes (0SRC, 0SRD, 0SWC, 0SWD, 0SR9, 0SRB, 0SW9, 0SWB). The presence of orthopaedic joint implants was confirmed using ICD‐10 code ‘Z96.6x’.

PJI cases were identified using ICD‐9‐CM code 996.66 in combination with associated procedure codes: (i) 80.05, (ii) 80.06, (iii) 81.53, (iv) 81.55, (v) 86.22 or 86.28 following THA, (vi) 86.22 or 86.28 following TKA or (vii) E878.1. For ICD‐10, PJI was identified using codes T84 or T81.4, along with specific procedure codes. Data on revision arthroplasty were obtained using relevant ICD‐9‐CM and ICD‐10‐AM codes for hip and knee prosthesis removal and revision (Supporting Information).

Infectious spondylitis was diagnosed using ICD‐9‐CM code 720.9, along with documentation of intravenous (IV) antibiotic use during the initial hospitalisation.

**Figure 1:**
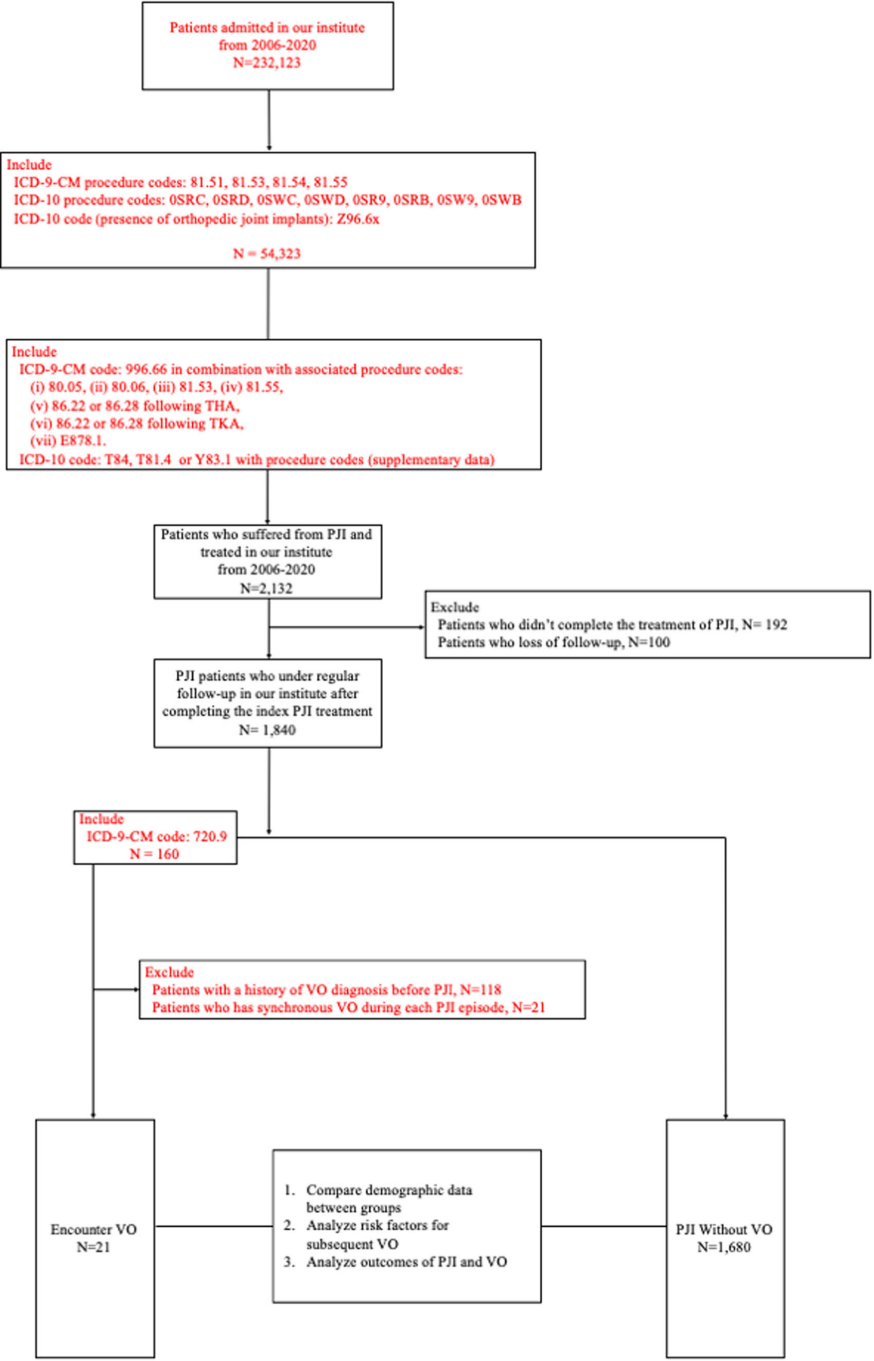
Patient selection and analysis flowchart. This figure outlines the process used to identify and analyse patients in our study conducted at our institute from 2006 to 2020. Starting with a base of 232,123 patients admitted during the study period, we narrowed our focus to 54,323 patients who underwent total hip arthroplasty (THA) or total knee arthroplasty (TKA) identified using specific International Classification of Diseases, Ninth Revision, Clinical Modification (ICD‐9‐CM) and ICD‐10 procedure codes. After further refinement based on ICD codes related to periprosthetic joint infection (PJI), we followed 1840 patients who completed their initial PJI treatment and were under regular follow‐up. The analysis included 21 patients who developed vertebral osteomyelitis (VO) post‐PJI and 1680 patients without VO. The study objectives were to compare demographic data, analyse risk factors for subsequent VO and assess the outcomes of PJI and VO.

The study population was identified from patients admitted to our institute between 2006 and 2020 (*N* = 232,123, Figure 1). From this population, patients who had undergone THA or TKA were identified using the aforementioned ICD‐9‐CM and ICD‐10 codes, resulting in a cohort of 54,323 patients.

PJI cases were further identified within this cohort using the specified ICD‐9‐CM and ICD‐10 codes. This resulted in a cohort of 2132 patients with PJI. Patients who did not complete the treatment of PJI (*N* = 192) and those who were lost to follow‐up (*N* = 100) were further excluded, leaving a final study population of 1840 PJI patients who were under regular follow‐up.

Furthermore, patients encountered VO (*N* = 160) were identified using ICD‐9‐CM 720.9. Among these, patients with a history of VO diagnosis before PJI (*N* = 118) or with synchronous VO during each PJI episode (*N* = 21) were excluded.

Finally, patients who encountered VO (*N* = 21) were identified. The remaining 1680 PJI patients without VO were also included in the study.

The study tracked patients until the endpoint of metachronous VO development. The analysis included comparing demographic data between groups, analysing risk factors for subsequent VO and analysing outcomes of PJI and VO.

### Definitions

In our study, we defined PJI using a combination of the following diagnostic criteria: (1) the presence of a sinus tract communicating with the prosthetic joint; (2) the isolation of a microorganism from two or more samples collected from the infected joint area or (3) at least four of the following six signs: elevated serum erythrocyte sedimentation rate (ESR) greater than 30 mm/h, elevated C‐reactive protein (CRP) greater than 10 mg/L, high leucocyte count in synovial fluid greater than 3000 cells/μL, increased percentage of neutrophils in synovial fluid greater than 80%, the presence of pus within the joint, a positive culture from tissue or fluid around the prosthesis and the observation of more than five neutrophils per high‐power field in at least five high‐power fields in histological samples of periprosthetic tissue at ×400 magnification [24].

### Outcome measures

In this study, we meticulously collected and analysed a range of patient data. This included demographic information, health condition histories, details of the arthroplasty (such as its specific site and date) and the approaches used to manage PJI. Additionally, we monitored the time lapse between the diagnosis of PJI and the onset of VO, the duration of antibiotic treatments administered and the types of pathogens identified in both PJI and VO cases. A comparative approach was employed to investigate the risk factors leading to VO in PJI patients, contrasting those who developed VO against those who did not. This comprehensive analysis facilitated a detailed understanding of risk factors and enabled us to accurately document the outcomes for these patients.

### Treatment

Patients with PJI underwent treatment adhering to the Tsukayama guidelines [22]. For acute hematogenous PJI cases, the protocol included debridement, irrigation and modular component replacement. Chronic infections, persisting beyond 4 weeks after symptoms appear, were primarily treated with a two‐stage resection arthroplasty, incorporating an interim antibiotic‐loaded bone cement (ALBC) spacer. This method commenced with preoperative blood cultures in cases of systemic inflammatory response syndrome (SIRS), followed by extensive debridement, removal of the prosthesis and ALBC placement. Postsurgery, patients underwent a 4‐week IV antibiotic regimen based on culture results, supplemented by a 2‐week oral course for cases with negative cultures. The ALBC used comprised 4 g each of vancomycin and ceftazidime per 40 g of cement. A 3‐month interval between resection stages was standard, including a 6‐week medication hiatus, with regular ESR and CRP monitoring. Reimplantation in the second stage was contingent upon normalised CRP levels and the absence of infection signs.

In the event of unsuccessful initial stage treatment, as indicated by on‐going positive joint cultures, elevated CRP or persistent symptoms of infection, we advise a repeated resection arthroplasty using a culture‐adapted ALBC spacer. The procedure for the subsequent stage is similar to the first, with the ALBC spacer adjusted according to the culture findings. Postoperative care involves a 4‐week course of IV antibiotics, followed by 2 weeks of oral antibiotics. The definition of repeat resection arthroplasty is based on the number of surgical interventions prior to the reimplantation, categorised as either three‐ or four‐stage procedures.

For recurrent PJI postreimplantation, the same principles apply, potentially leading to another two‐stage resection arthroplasty, termed ‘repeat 2‐stage resection arthroplasties’. Recurrent PJI complications can include scar formation, soft tissue contraction, resistant infections and in severe cases, septic shock, necessitating amputation or permanent resection arthroplasty as life‐saving measures [16].

Polymicrobial PJI, defined as isolating multiple microorganisms from periprosthetic tissue or synovial fluid cultures, was also studied for its microbiological profile.

In our research, VO was diagnosed by assessing symptoms such as back pain, alongside elevated levels of ESR and CRP. Magnetic resonance imaging was the primary tool for definitive diagnosis, with confirmation from spine surgeons. When additional tissue samples were required, computed tomography‐guided biopsy or debridement surgery was employed.

At our facility, the treatment approach for VO typically begins with conservative management, primarily involving prolonged antibiotic therapy. Surgical intervention is considered for those cases where antibiotic treatment proves ineffective and in scenarios involving neurological complications, structural instability of the spine, continuous back pain or the presence of an epidural abscess.

### Statistical analysis

To assess the relationship between qualitative variables, we employed the *χ* ^2^ test or Fisher's exact test as appropriate. For continuous variables that followed a normal distribution, we used analysis of variance for comparisons. Risk factor evaluation was conducted through both univariate and multivariate logistic regression models. A *p* value of less than 0.05 (5%) was considered statistically significant for all tests conducted. The data processing and analysis were carried out using Statistical Product and Service Solutions software (version 20.0, IBM Corp.).

## RESULTS

In our cohort of 1701 PJI patients, with a minimum of 5 years of clinical follow‐up, 21 individuals (1.23%, 21/1701) developed metachronous VO. The gender distribution among these patients was 57.1% male (12 patients) and 42.9% female (nine patients), with an average age of 70.3 years (standard deviation: 11.1 years).

We compared demographic data between patients who did and did not develop metachronous VO (refer to Table 1). To determine risk factors, we utilised logistic regression modelling, taking into account various factors such as patient comorbidities, PJI treatment methods, intraoperative details and identified causative pathogens. Key risk factors for metachronous VO post‐PJI were found to be SIRS, history of substance abuse, polymicrobial nature of PJI and undergoing three or more stages of resection arthroplasty (odds ratios: 1.86, 54.28, 52.33 and 31.88, respectively), as detailed in Table 2.

**Table 1:** Demographic comparison between patients with metachronous VO and those without.

Parameters	PJI with metachronous VO (*n* = 21)	PJI without metachronous VO (*n* = 1701)	*p*
Fundamental information			
Gender distribution (male/female)	12(57.1%)/9(42.9%)	993(58.4%)/708(41.6%)	0.941
Average age (SD)	70.3 (11.1)	70.816 (11.2)	0.961
Mean body mass index (SD)	24.1 (3.3)	24.3 (3.2)	0.549
Serum albumin level (SD)	2.6 (0.6)	2.8 (0.6)	0.061
Estimated glomerular filtration rate (SD)	65.2 (32.1)	71.2 (34.2)	0.597
C‐reactive protein level (SD)	100.1 (67.1)	91.8 (83.7)	0.469
Clinical hospitalisation details			
Admittance via emergency room	13 (61.9)	201 (11.8)	<0.001*
Presence of systemic inflammatory response syndrome	15 (71.4)	203 (11.9)	<0.001*
Positive results in blood cultures	12 (57.1)	69 (4.1)	<0.001*
Pre‐existing medical conditions			
Charlson comorbidity index score	3.5 (2.3)	2.4 (2.1)	0.163
Prevalence of cancer (%)	6 (28.6)	171 (10.1)	0.132
Incidence of solid tumours (%)	3 (14.3)	78 (5.1)	0.123
Proportion with hypertension (%)	15 (71.4)	1092 (64.2)	0.705
Diabetes incidence (%)	9 (42.9)	291 (17.1)	0.101
Occurrence of liver disease (%)	9 (42.9)	459 (27.0)	0.123
Hepatitis C virus carriers (%)	9 (42.9)	108 (6.4)	0.012*
Cases of alcoholism (%)	3 (14.3)	75 (4.4)	0.123
Instances of drug use (%)	6 (28.6)	45 (2.6)	<0.001*
Renal insufficiency frequency (%)	3 (14.3)	96 (5.6)	0.380
Cardiovascular disease prevalence (%)	9 (42.9)	291 (17.1)	0.123
Atrial fibrillation (%)	3 (42.9)	48 (2.8)	<0.001*
Coronary artery disease (%)	1 (14.3)	72 (4.2)	0.256
Surgical procedure variables			
Duration of surgery (min) (SD)	135 (40.2)	133 (48.1)	0.129
Affected joint			
Hip involvement (%)	3 (14.3)	482 (28.3)	0.123
Knee involvement (%)	18 (85.7)	1219 (71.7)	0.113
Surgical techniques			
Two‐stage resection arthroplasty with mobile spacer (%)	4 (19.0)	1407 (82.7)	0.092
Two‐stage resection arthroplasty with static spacer (%)	7 (33.3)	110 (6.4)	0.012*
Three‐stage or more resection arthroplasty (%)	6 (28.6)	46 (2.7)	0.003*
Debridement, antibiotics and implant retention (%)	4 (19.0)	165 (9.7)	0.136
Necessity of amputation (%)	3 (14.3)	52 (3.1)	0.576
Repeat two‐stage resection arthroplasty (%)	11 (52.4)	91 (5.3)	0.023*
Pathogens in PJI			
Cases without cultured pathogens (%)	10 (42.6)	385 (22.6)	0.242
Gram‐positive bacterial infections (%)	9 (42.9)	1181 (69.4)	0.089
Gram‐negative bacterial infections (%)	1 (4.8)	119 (7.0)	0.465
Fungal infections (%)	5 (23.8)	46 (2.7)	0.003*
Tuberculosis infections (%)	1 (4.8)	22 (1.29)	0.750
Incidence of polymicrobial infections (%)	8 (38.1)	71 (4.2)	<0.001*
Methicillin‐resistant	8 (38.1)	140 (8.2)	0.098
*Staphylococcus aureus* (%)			

Abbreviations: PJI, periprosthetic joint infection; VO, vertebral osteomyelitis.

* *p* Value < 0.05.

**Table 2:** Outcomes from multivariate logistic regression on factors linked to metachronous VO.

	Multivariate Model results	
Factors	Adjusted OR (95% CI)	*p*
Emergency room admissions	0.38 (0.72–1.34)	0.321
Systemic inflammatory response syndrome presence	1.86 (1.23–1.91)	<0.001*
Positive blood cultures	0.82 (0.79–1.06)	0.248
Hepatitis C virus carriage	3.21 (0.45–66.41)	0.324
Substance abuse cases	54.28 (9.12–78.21)	0.003*
Incidence of atrial fibrillation	2.32 (0.70–28.83)	0.234
Two‐stage resection arthroplasty (using static spacer)	7.21 (0.41–99.90)	0.221
Resection arthroplasty of three or more stages	31.88 (8.29–97.45)	<0.001*
Repeat of two‐stage resection arthroplasty	8.14 (0.73–87.23)	0.323
Fungal infections	13.6 (0.77–117.95)	0.218
Polymicrobial PJI	52.33 (7.31–115.33)	<0.001*

Abbreviations: CI, confidence interval; OR, odds ratio; PJI, periprosthetic joint infection; VO, vertebral osteomyelitis.

* *p* Value < 0.05.

Among the 21 patients who developed metachronous VO subsequent to PJI, we observed several significant clinical outcomes, as detailed in Table 3. A majority, 18 patients (85.7%), experienced SIRS. Blood culture results were positive in 15 patients (71.4%), while 12 patients (57.1%) had negative tissue cultures. Recurrent infections were seen in 18 patients (85.7%), who subsequently underwent numerous complex debridement surgeries. Notably, nine patients (42.9%) developed metachronous VO within a month following their PJI diagnosis, all presenting with negative tissue cultures.

**Table 3:** Notable observations in individuals who developed VO sequentially after experiencing a PJI.

Case	Blood culture with positive results	SIRS	Procedures	Affected area	Infection date	Duration from prosthetic joint infection to VO (days)	Recurrent infection	Pathogen	Same species	Final outcomes
1	+	+	Three‐stage resection arthroplasty	Right hip	20 November 2012	280	Yes (2)	*Candida metapsilosis*	‐	Right hip disarticulation
								*Staphylococcus epidermidis*		
				L4–S1	27 August 2013		0	*M. osloensis*		Transforaminal lumbar interbody debridement and fusion
2	+	+	Three‐stage resection arthroplasty	Right knee	10 June 2006	183	0	*Staphylococcus aureus*	‐	Functioning effectively without recurring infections
				L3–L4	10 December 2006		0	No growth		Functioning effectively without recurring infections
3	‐	‐	Three‐stage resection arthroplasty	Left knee	6 November 2012	889	Yes (2)	No growth	‐	Functioning effectively without recurring infections
				T10–12	14 April 2015		0	No growth		Transforaminal thoracic interbody debridement and fusion
4	+	+	Two‐stage with mobile spacer	Left knee	1 March 2014	242	Yes (2)	*Streptococcus agalactiae*	‐	Permanent articular mobile spacer without reimplantation
				L2–L4	29 October 2014		Yes (2)	No growth		TLIDF with recurrent VO
5	‐	+	Two‐stage with mobile spacer	Right knee	7 April 2013	738	Yes (4)	No growth	‐	Functioning effectively without recurring infections
				L3–L4	15 April 2015		Yes (2)	*K. pneumoniae*		Transforaminal lumbar interbody debridement and fusion
6	+	+	Two‐stage with mobile spacer	Left knee	8 May 2013	153	Yes (4)	No growth	‐	Permanent articular mobile spacer without reimplantation
				L3–L4	8 October 2013		0	No growth		Transforaminal lumbar interbody debridement and fusion
7	+	+	Three‐stage resection arthroplasty	Left knee	28 September 2011	361	Yes (3)	*S. agalactiae*	+	Permanent articular mobile spacer without reimplantation
				L4–L5	23 September 2012		0	*S. agalactiae*		Transforaminal lumbar interbody debridement and fusion
8	+	+	Three‐stage resection arthroplasty	Right hip	10 October 2010	293	Yes (3)	*C. metapsilosis*	‐	Right hip disarticulation
								*K. pneumoniae*		
				L2–L3	31 July 2011		0	*S. agalactiae*		Transforaminal lumbar interbody debridement and fusion
9	+	+	Three‐stage resection arthroplasty	Left knee	10 September 2008	60	0	*Staphylococcus aureus*	‐	Functioning effectively with antibiotic suppression
				L3–L4	9 November 2008		0	*Candida Metapsilosi*		Functioning effectively with antibiotic suppression
10	‐	+	Two‐stage with mobile spacer	Left knee	7 April 2013	736	Yes (2)	No growth	‐	Functioning effectively with antibiotic suppression
				L4–L5	13 April 2015		Yes (3)	*K. pneumoniae*		Transforaminal lumbar interbody debridement and fusion
11	‐	‐	Three‐stage resection arthroplasty	Right knee	6 May 2012	1071	Yes (3)	*S. epidermidis*	‐	Functioning effectively with antibiotic suppression
				T12–L2	12 March 2015		0	No growth		Transforaminal thoracic and lumbar interbody debridement and fusion
12	+	+	Two‐stage with mobile spacer	Left knee	11 February 2013	493	Yes (3)	*S. agalactiae*	‐	Permanent articular mobile spacer without reimplantation
				L1–L4	19 June 2014		Yes (2)	No growth		Transforaminal lumbar interbody debridement and fusion
13	+	+	Two‐stage with mobile spacer	Right knee	18 March 2012	445	Yes (2)	No growth	‐	Permanent articular mobile spacer without reimplantation
				L2–L4	6 June 2013		0	No growth		Transforaminal lumbar interbody debridement and fusion
14	+	+	Three‐stage resection arthroplasty	Right knee	28 July 2010	601	Yes (2)	*C. metapsilosis*	+	Permanent articular static spacer without reimplantation
				L2–L3	20 March 2012		0	*S. agalactiae*		Transforaminal lumbar interbody debridement and fusion
15	+	+	Three‐stage resection arthroplasty	Left hip	10 October 2011	683	Yes (3)	*C. metapsilosis*	‐	Left hip disarticulation
				L4–S1	23 August 2013		0	*M. osloensis*		Transforaminal lumbar interbody debridement and fusion
16	+	+	Three‐stage resection arthroplasty	Left knee	13 March 2006	645	0	*S. aureus* *S. agalactiae*	‐	Functioning effectively without recurring infections
				L1–L2	18 December 2007		0	No growth		Functioning effectively without recurring infections
17	‐	+	Two‐stage with mobile spacer	Left knee	17 April 2012	1131	Yes (4)	No growth	‐	Functioning effectively without recurring infections
				L3–L4	23 May 2015		Yes (3)	*K. pneumoniae*		Transforaminal lumbar interbody debridement and fusion
18	‐	‐	Three‐stage resection arthroplasty	Left knee	6 October 2011	495	Yes (2)	No growth	‐	Revision arthroplasty
				T11–12	12 February 2013		0	No growth		Transforaminal thoracic interbody debridement and fusion
19	+	+	Two‐stage with mobile spacer	Right knee	11 August 2013	312	Yes (2)	*S. agalactiae*	‐	Permanent articular mobile spacer without reimplantation
				L2–L4	19 June 2014		0	No growth		Transforaminal lumbar interbody debridement and fusion
20	+	+	Two‐stage with mobile spacer	Rightt knee	18 February 2012	879	Yes (2)	No growth	‐	Permanent articular mobile spacer without reimplantation
				L3–L4	16 July 2014		0	No growth		Transforaminal lumbar interbody debridement and fusion
21	+	+	Three‐stage resection arthroplasty	Left knee	18 October 2013		Yes (3)	*S. agalactiae*	+	Permanent articular mobile spacer without reimplantation
				L3–L5	11 September 2014		0	*S. agalactiae*		Transforaminal lumbar interbody debridement and fusion

Abbreviations: PJI, periprosthetic joint infection; SIRS, systemic inflammatory response syndrome; TLIDF, transforaminal lumbar interbody debridement and fusion; VO, vertebral osteomyelitis.

## DISCUSSION

This retrospective study delved into potential risk factors linking PJI with metachronous VO, focusing on a history of SIRS, drug abuse, polymicrobial PJI and instances of three or more stages of resection arthroplasty. Known risk factors for PJI and VO encompass patient conditions like advanced age, specific comorbidities, substance abuse and prior surgeries [5]. Pathogens such as *Staphylococcus aureus*, coagulase‐negative staphylococci and others have been identified as causative agents in both conditions [20]. Despite potential overlaps, the correlation between these two diseases has not been extensively explored. Our study excluded cases of synchronous PJI to isolate the development of metachronous VO as a distinct phenomenon.

Bacteremia is hypothesised as a possible pathway for metachronous VO following PJI, emphasising the need for clinicians to specifically evaluate for this condition [6]. Bacteremia detection rates, which can vary based on prior antibiotic treatment, play a crucial role in this context [2, 15]. Clinical procedures, including dental work, can escalate the risk of bacteremia, further complicating the clinical scenario [21]. However, studies focusing on bacteremia in PJI patients are limited and the actual rates may be underestimated due to diagnostic criteria that do not mandate blood cultures and the potential for false positives [3, 17]. In our study, despite a high rate of positive blood cultures (71.4%), establishing a direct link to metachronous VO development remains challenging.

Our research also emphasises the role of the patient's clinical condition, particularly, SIRS and substance abuse, in the onset of metachronous VO following PJI. A significant portion of our cohort presented with SIRS during PJI, identifying it as an independent risk factor. Substance abuse, particularly, during PJI treatment, emerged as a major risk factor. The International Consensus Group on Periprosthetic Joint Infection recommends postponing arthroplasty in patients with substance abuse history [1]. These patients are more prone to bacteremia due to their compromised immunity [10].

Polymicrobial PJIs, though less common, present significant risks in hip and knee arthroplasty patients [12]. These infections, often involving virulent pathogens, necessitate the use of broad‐spectrum antibiotics [25]. Our data shows that polymicrobial PJI patients undergoing a two‐stage resection arthroplasty face poorer outcomes, potentially increasing their susceptibility to bacteremia and consequently, metachronous VO.

Within our study group, a significant majority of 85.7% (18 out of 21 patients) who developed metachronous VO had previously experienced PJIs in the knee joint. This observation suggests that knee PJIs might have a higher treatment failure rate, potentially leading to an increased likelihood of bacteremia, which, in turn, could contribute to the development of metachronous VO in patients at higher risk. Nevertheless, the precise mechanisms underlying this association remain unclear and warrant further investigation, including genetic analyses of bacteria isolated from PJI, blood and VO samples.

In our research, we categorised patients with metachronous VO into two groups: those with a short interval (VO development within one month of PJI diagnosis) and those with a long interval post‐PJI. For the short‐interval group, bacteremia is speculated to be a potential cause. However, the prevalence of negative cultures in spinal tissue samples hinders definitive conclusions regarding aetiology. These negative cultures might be attributed to the extensive antibiotic treatment employed in our PJI management protocols. On the other hand, the clinical trajectories of long‐interval cases were more amenable to analysis. These patients typically experienced recurrent PJIs with variable CRP levels during their treatment. The prolonged antibiotic therapy in these cases likely contributed to the difficulty in culturing pathogenic bacteria. Consequently, obtaining definitive tissue cultures in cases of both short‐ and long‐interval metachronous VO was challenging. Additionally, the lack of genetic analysis data for most of the study period prevented us from determining whether the bacteria causing PJI, blood infections and spinal infections were the same.

Our study is subject to several limitations, primarily due to its retrospective case–control nature. This design inherently carries the risk of selection bias and incomplete data. Efforts were made to mitigate bias by consistently applying uniform treatment protocols and rehabilitation programmes across the study. However, a more comprehensive evaluation of clinical outcomes would benefit from extended follow‐up periods. Additionally, the conclusions of our study should be interpreted with caution due to the small sample size in one of the study arms, which makes it susceptible to bias and limits the generalisability of the findings.

## CONCLUSIONS

In our research, we found the occurrence of metachronous VO post‐PJI to be 1.2%. Key risk factors contributing to VO development were identified as the occurrence of SIRS during PJI, a history of substance abuse, the presence of polymicrobial PJI and undergoing multiple (three or more) stages of resection arthroplasty.

Given these findings, we suggest that surgeons consider closer monitoring and more aggressive management strategies for patients with PJI who present with these risk factors. Early identification and intervention could potentially reduce the incidence of metachronous VO. This might include prolonged antibiotic therapy, regular follow‐up imaging and early consideration of surgical intervention in cases where VO is suspected.

The pathogenesis and clinical progression of this condition warrant further exploration, particularly, through prospective studies that include genetic analysis of the pathogens involved. Future research should focus on developing targeted strategies to prevent VO in high‐risk patients.

## AUTHOR CONTRIBUTIONS

**Yu‐Chih Lin**: Conceptualisation; methodology; software; writing—original draft preparation. **An‐Jhih Luo**: Data curation. **Fu‐Cheng Kao**: Supervision. **An‐Shun Tai**: Software. **Yuhan Chang**: Investigation. **Pang‐Hsin Hsieh**: Validation. **Sheng‐Hsuan Lin**: Writing—reviewing and editing. **Sheng‐Hsun Lee**: Conceptualization and methodology reviewing.

## CONFLICT OF INTEREST STATEMENT

The authors declare no conflict of interest.

## ETHICS STATEMENT

Our study involved the comparison and analysis of de‐identified, population‐level data. Our study was approved by the Institutional Review Board (IRB: 201601034B0). The study was performed at Chang Gung Memorial Hospital (CGMH). Informed consent was waived due to the retrospective nature of the study. The authors affirm that human research participants provided informed consent for the publication of the images in Figure 1 and Tables 1, 2, 3.

## Supporting information

Supporting information.

## Data Availability

The data, materials and/or Code generated during and/or analysed during the current study are available from the corresponding author on reasonable request.
