# Supplementary material for: Key risk factors and adverse outcomes in metachronous vertebral osteomyelitis following periprosthetic joint infection: A 5‐year retrospective study
Source: J Exp Orthop. 2024 Jul 5;11(3):e12083. doi: 10.1002/jeo2.12083 (PMC11224968; doi:10.1002/jeo2.12083)
Supplement: Supplementary file 1 — Supporting information. [file JEO2-11-e12083-s001.docx]

| **Definition of outcomes** | |
| --- | --- |
| **Total knee arthroplasty (TKA)** | ICD-9 procedure code: 81.54  ICD-10 procedure code: 0SRC~(right knee joint)、0SRD~(left knee joint)、49518–00: Total arthroplasty of knee, unilateral、49519–00: Total arthroplasty of knee, bilateral |
| **Revision of TKA** | ICD-9 procedure code: 81.55, 80.06  ICD-10 diagnosis code: Z96.6x—Presence of orthopedic joint implants  ICD-10 procedure code:  0SWC~(right knee joint)、0SWD~(left knee joint)、49527-00: Revision of total arthroplasty of knee (includes removal of prosthesis)、49312-00: Excision arthroplasty of knee (removal of prosthesis without replacement) includes insertion of cement spacer |
| **Total hip arthroplasty (THA)** | ICD-9 procedure code: 81.51  ICD-10 procedure code: 0SR9~(right hip joint)、0SRB~(left hip joint)、49318-00: Total arthroplasty of hip, unilateral、49319-00: Total arthroplasty of hip, bilateral |
| **Revision of THA** | ICD-9 procedure code: 81.53, 80.05  ICD-10 diagnosis code: Z96.6x—Presence of orthopedic joint implants  ICD-10 procedure code: 0SW9~(right hip joint)、0SWB~(left hip joint)、49324-00: Revision of total arthroplasty of hip (includes removal of prosthesis)、49312-00: Excision arthroplasty of knee (removal of prosthesis without replacement) includes insertion of cement spacer |
| **Periprosthetic joint infection (PJI)** | ICD-9 diagnostic code of 996.66 and procedure codes of 1) 80.05, 2) 80.06, 3) 81.53, 4) 81.55, 5) 86.22 or 86.28 in one month after THA, 6) 86.22 or 86.28 in one month after TKA, or 7) E878.1: Surgical operation with implant of artificial internal device  ICD-10 diagnostic codes starting with T84 or T81.4 combined with below procedure codes or Y83.1: Surgical operation with implant of artificial internal device.   1. T84.51 Infection and inflammatory reaction due to internal right hip prosthesis; 2. T84.52 Infection and inflammatory reaction due to internal left hip prosthesis; 3. T84.53 Infection and inflammatory reaction due to internal right knee prosthesis; 4. T84.54 Infection and inflammatory reaction due to internal left knee prosthesis combined with procedure codes of 0SP909Z、0SP90JZ、0SP93JZ、0SP94JZ、0SPA0JZ，0SPA3JZ、0SPA4JZ、0SPB09Z、0SPB0JZ、0SPB3JZ、0SPB4JZ、0SPE0JZ、0SPE3JZ、0SPE4JZ、0SPR0JZ、0SPR3JZ、0SPR4JZ、0SPS0JZ、0SPS3JZ、0SPS4JZ、0SPC09Z、0SPC0JC、0SPC0JZ、0SPC3JC、0SPC3JZ、0SPC4JC、0SPC4JZ、0SPD09Z、0SPD0JC、0SPD0JZ、0SPD3JC、0SPD3JZ、0SPD4JC、0SPD4JZ、0SPT0JZ、0SPT3JZ、0SPT4JZ、0SPU0JZ、0SPU3JZ、0SPU4JZ、0SPV0JZ、0SPV3JZ、0SPV4JZ、0SPW0JZ、0SPW3JZ、0SPW4JZ、0SW90JZ、0SW93JZ、0SW94JZ、0SWA0JZ、0SWA3JZ、0SWA4JZ、0SWB0JZ、0SWB3JZ、0SWB4JZ、0SWE0JZ、0SWE3JZ、0SWE4JZ、0SWR0JZ、0SWR3JZ、0SWR4JZ、0SWS4JZ、0SWS3JZ、0SWS0JZ、0SWC0JC、0SWC0JZ、0SWC3JC、0SWC3JZ、0SWC4JC、0SWC4JZ、0SWD0JC、0SWD0JZ、0SWD3JC、0SWD3JZ、0SWD4JC、0SWD4JZ、0SWT0JZ、0SWT3JZ、0SWT4JZ、0SWU0JZ、0SWU3JZ、0SWU4JZ、0SWV0JZ、0SWV3JZ、0SWV4JZ、0SWW0JZ、0SWW3JZ、0SWW4JZ、0HBHXZZ、0HBJXZZ、0HBKXZZ、0HBLXZZ、0JBC0ZZ、0JBL0ZZ、0JBM0ZZ、0JBN0ZZ、0JBP0ZZ、0HDDXZZ、0HDEXZZ、0HDHXZZ、0HDJXZZ、0HDKXZZ、0HDLXZZ、0JDG0ZZ、0JDH0ZZ、0JDL0ZZ、0JDM0ZZ、0JDN0ZZ、0JDP0ZZ、0KDN0ZZ、0KDP0ZZ、0KDQ0ZZ、0KDR0ZZ、0KDS0ZZ、0KDT0ZZ、0LDJ0ZZ、0LDK0ZZ、0LDL0ZZ、0LDM0ZZ、0LDN0ZZ、0LDP0ZZ、0LDQ0ZZ、0LDR0ZZ、0QD20ZZ、0QD30ZZ、0QD40ZZ、0QD50ZZ、0QD60ZZ、0QD70ZZ、0QD80ZZ、0QD90ZZ、0QDB0ZZ、0QDC0ZZ、0QDD0ZZ、0QDF0ZZ、0QDG0ZZ、0QDH0ZZ、0QDJ0ZZ、0QDK0ZZ、3E1038Z、3E10X8Z |
